# Association transient receptor potential melastatin channel gene polymorphism with primary open angle glaucoma

**Published:** 2013-08-24

**Authors:** Seydi Okumus, Seniz Demiryürek, Bülent Gürler, Erol Coskun, İbrahim Bozgeyik, Serdar Oztuzcu, Erdal Kaydu, Oguz Celik, İbrahim Erbagcı, Abdullah T. Demiryürek

**Affiliations:** 1 Department of Ophthalmology, Faculty of Medicine, University of Gaziantep, Gaziantep, Turkey; 2 Department of Physiology, Faculty of Medicine, University of Gaziantep, Gaziantep, Turkey; 3 Department of Medical Biology, Faculty of Medicine, University of Gaziantep, Gaziantep, Turkey; 4 Department of Medical Pharmacology, Faculty of Medicine, University of Gaziantep, Gaziantep, Turkey

## Abstract

**Purpose:**

Genetic factors are shown to have a role in the development of primary open angle glaucoma (POAG). The aim of this study was to determine the effects of genetic polymorphisms of transient receptor potential melastatin (TRPM) channel genes on the risk of POAG in a Turkish population.

**Methods:**

Genomic DNA was extracted from the leukocytes of the peripheral blood, and 26 single nucleotide polymorphisms in the TRPM channel genes were analyzed in 179 patients with POAG and in 182 healthy controls of similar age by using the BioMark HD dynamic array system.

**Results:**

There were marked changes in the genotype (TT, 26.8%; CT, 66.7%; CC, 6.5%) and allele (T, 60.1%; C, 39.9%) frequencies for the *TRPM5* gene rs34551253 (Ala456Thr, in exon 9) polymorphism in patients when compared to the controls (TT, 11.3%; CT, 74.6%; CC, 14.1%, p=0.0009; T, 48.6%; A, 51.4%, p=0.0063). However, no associations with the other 25 polymorphisms studied were found.

**Conclusions:**

This is the first study to examine the involvement of *TRPM* channel gene variations in the risk of incident POAG. This study demonstrated that the *TRPM5* gene rs34551253 (Ala456Thr) polymorphism may be associated with increased risk of developing POAG in the Turkish population.

## Introduction

Glaucoma is a complex and heterogeneous disease characterized by a progressive degeneration of the axons of the retinal ganglion cells (RGCs), but the mechanisms causing the RGC loss are still undetermined. Glaucoma is the second most common cause of blindness worldwide [1]. Primary open angle glaucoma (POAG), the most common form of glaucoma, is a complex chronic degenerative disease with a multifactorial etiology including mechanical damage due to eleveted intraocular pressure (IOP), increased glutamate levels, mutations in specific genes, toxic effects and oxidative damage caused by reactive oxygen species (ROS) [2], and apoptosis [3]. Elevation of IOP also causes retinal ischemia and neuronal cell death. However, most of the molecular mechanisms leading to POAG development are still unknown. Several genetic loci that contribute to the susceptibility of eyes to POAG have been identified. Genome-wide association studies showed that only two identified genes, myocilin (*MYOC*) and optineurin (*OPTN*), are well-established glaucoma-causing genes [4].

Transient receptor potential (TRP) channels are a highly diverse group of ion channels with widely diverging functional properties. Various stimuli can regulate the gating of the TRP channels, such as physical stimuli (temperature, voltage, mechanical stress), exogenous ligands, intracellular cations, and lipid components of the plasma membrane [5]. There are 28 mammalian TRP channels, which are subdivided into six subfamilies based on protein sequence homology: TRPA (ankyrin), TRPC (canonical), TRPM (melastatin), TRPML (mucolipin), TRPP (polycystin), and TRPV (vanilloid) [6]. The TRPM subfamily consists of eight members (TRPM1–8). Three members of the TRPM subfamily (TRPM2, TRPM6, and TRPM7) have enzymatic activity. Moreover, TRPM4 and TRPM5 are impermeable to Ca^2+^ [6]. TRPM1 is the founding member of the TRPM subfamily of TRP channels, and was initially identified by differential display as a potential suppressor of tumor metastasis (and originally named melastatin because it is downregulated in metastatic melanoma) [7]. TRPM2 forms a non-selective cation channel gated by adenosine diphosphate-ribose, hydrogen peroxide, and intracellular Ca^2+^ [8,9]. TRPM3 is activated by sphingolipids [9], and shown to function in insulin secretion [10]. TRPM4 controls pressure-mediated smooth muscle cell depolarization and vasoconstriction of cerebral arteries, so it may participate in autoregulating cerebral blood flow [11,12]. TRPM5 is activated by Ca^2+^ released from intracellular stores, and has a role in taste transduction [13]. TRPM6 and TRPM7 are close members of the TRP superfamily of ion channels with ion channel and protein kinase activities and have been shown to play important roles in magnesium homeostasis. TRPM6 and TRPM7 are highly permeable to magnesium [14,15]. TRPM8 is involved in thermosensation [16]. However, the role of TRPM channels in POAG is not known.

TRPM1 is a visual transduction channel in retinal ON bipolar cells. Four recent articles reported an association between mutations in the TRPM1 gene and autosomal recessive congenital stationary night blindness in humans [17-20]. In the complete form of congenital stationary night blindness, various mutations, including deletions and nonsense mutations, leading to loss of function were found. Since TRPM channels are present in the vascular and ocular cells, vasoconstriction may lead to optic nerve head ischemia and subsequent development of visual field defects during the course of glaucoma; we hypothesize that TRPM channels gene variants play a role in the risk of POAG development. The purpose of the present study was to investigate a possible association between TRPM gene polymorphisms and POAG in a Turkish population.

## Methods

### Study population

A total of 179 unrelated Turkish patients (91 males and 88 females; age range, 33-79 years old) and 182 healthy controls (93 males and 89 females; age range, 29-80 years old) evaluated at University of Gaziantep, Faculty of Medicine, Department of Ophthalmology, Gaziantep, Turkey were recruited into this study. Routine ophthalmologic evaluations were performed on all subjects. Patients with POAG underwent a complete eye examination including visual acuity testing, slit-lamp biomicroscopy with and without dilation, fundus examination with 90-diopter lens, IOP measurement with Goldmann applanation tonometry, central corneal thickness (CCT) measurement with ultrasonic pachymetry, and gonioscopy. The criteria for classifying a patient as having POAG were as follows: IOP>22 mmHg in each eye without antiglaucoma drugs, pathological cupping of the optic disc including the quantitative assessment of cup-to-disc (C/D) ratio >0.7 in each eye, visual field defects determined with automated perimetry (Octopus 900, Haag-Streit, Switzerland) and/or Humphrey visual field analysis consistent with the glaucomatous cupping in at least 1 eye, and an open anterior chamber angle. We checked the IOP on at least three visits, and the measurements were made during the daylight hours. Patients with a history of eye surgery before the diagnosis of glaucoma, or with evidence of secondary glaucoma, such as exfoliation, pigment dispersion, trauma-, uveitis-, or steroid-induced glaucoma, were excluded. Patients with malignant or autoimmune diseases were also excluded. A control group of age- and sex-matched individuals was chosen randomly from a sample of patients admitted to the ophthalmology outpatient department for refractive errors, conjunctivitis, blepharitis, burning, itching, presbyopia, senile cataracts, routine ophthalmic examinations, or medical staff with no ocular problems. The control subjects had the following characteristics: IOP<22 mmHg, normal optic discs, with a C/D ratio of ≤0.3 and glaucoma hemifield test within normal limits, and no family history of glaucoma. The study was approved by the local Ethics Committee, in compliance with the Declaration of Helsinki.

### Blood samples and deoxynucleic acid isolation

Peripheral venous blood samples (5 ml) were obtained and collected in sterile siliconized vacutainer tubes with 2 mg/ml disodium EDTA. Immediately after collection, all samples were stored at −20 °C until use. Genomic DNA was extracted from whole blood with the salting-out method and stored at −20 °C.

### Genotyping

The genotype was determined in all patients and controls with the Fluidigm dynamic array system. Polymorphisms were analyzed in genomic DNA with a 96.96 dynamic array on the BioMark HD system (Fluidigm, South San Francisco, CA). Digital PCR Analysis software (Fluidigm) was used to process the data after the reaction. Chambers that yielded signals were detected and counted.

The following criteria were used to choose the single nuclear polymorphisms (SNPs): (1) relatively high minor allele frequencies in Caucasian populations; (2) location within the promoter region, and exonic and intronic sites that could potentially impact TRPM expression and function; and (3) suitable for the Fluidigm dynamic array chip designing, i.e., with no high G/C levels. In the present study, 26 SNPs [TRPM1: rs28441327 in intron 1, rs11070811 in intron 1, rs2241493 (Ser32Asn) in exon 5; TRPM2: rs1618355 in intron 18, rs9978351 (Arg1189=) in exon 24; TRPM3: rs113652718 (Val104Ala) in exon 6, rs1328142 in intron 9; TRPM4: rs3760663 in 5′-untranslated region, rs71352737 (Trp525Ter) in exon 11; TRPM5: rs34364959 (Gly900Ser) in exon 18, rs4929982 (Arg578Gln) in exon 11, rs34551253 (Ala456Thr) in exon 9, rs886277 (Asn235Ser) in exon 5, rs3986599 (Val254Ala) in exon 6; TRPM6: rs3750425 (Val1388Ile) in exon 28, rs2274924 (Lys1579Glu) in exon 29; TRPM7: rs77165588 in promoter; TRPM8: rs1016062 in intron 21, rs2362294 in intron 21, rs6431648 in intron 1, rs2362295 in intron 22, rs10803666 in intron 2, rs10490018 in intron 24, rs2052029 in 3′-untranslated region, rs2215173 in intron 7, and rs6740118 in intron 9] were studied for *TRPM* gene polymorphisms.

### Statistical analyses

Results are expressed as mean±standard deviation (SD) or percentage. Differences in genotype and allele frequencies among the cases and controls were tested with the chi-square test for independence and the chi-square test with Yate’s correction or Fisher’s exact tests. For comparisons of the differences between the mean values of two groups, the unpaired Student *t* test was used. The original significance level was set at a p value of 0.05. All probability values were based on two-tailed tests. To conclude the association, we used the Bonferroni method to correct the p values for multiple testing, using a stringent threshold. For correcting p values in model-based analysis, including the allele and full model, a p value of <0.0019 (0.05/26) was considered statistically significant. The odds ratio (OR) and 95% confidence intervals (CIs) were also calculated using logistic regression analysis. The SPSS statistical package (SPSS, version 17.0, Chicago, IL) and GraphPad Instat version 3.05 (GraphPad Software, San Diego, CA) were used for statistical analysis.

## Results

Clinical characteristics of the study population are presented in Table 1. In this study, 179 patients with POAG admitted to the ophthalmology clinic were investigated. The mean age and gender of the patients and control groups were similar. In glaucoma group, the mean IOP was 28.6±6.2 mmHg within a range between 22 and 55 mmHg, and the C/D ratio was 0.7±0.2. The mean CCT value was 557.8±37.7 μm within a range between 470 and 670 μm.

**Table 1:** Clinical characteristics of the study population.

Parameters	Controls	Cases with POAG	P value
	(n=182)	(n=179)	
Age (years)^a^	51.3±11.2	53.2±8.4	0.0694
**Gender**			
Male (n, %)	93 (51.1)	91 (50.8)	0.9605
Female (n, %)	89 (48.9)	88 (49.2)	
IOP (mmHg)a	13.3±3.4	28.6±6.2	**<0.0001**

^a^Data are mean±SD. POAG, primary open-angle glaucoma; IOP, intraocular pressure.

Table 2 and Table 3 show the distributions of the genotypes and alleles between the case and control groups. There were marked associations for the genotype (p=0.0009) frequencies of the *TRPM5* gene rs34551253 (Ala456Thr) polymorphism between the patients and the control group. The TT genotype frequency (26.8%) of this polymorphism was significantly higher in patients with POAG than in the controls (TT, 11.3%). We performed a logistic regression analysis model for the association between the minor allele of *TRPM5* rs34551253 (Ala456Thr) polymorphism and POAG. The TT genotype was significantly associated as a risk factor for POAG (OR: 4.93; 95% CI=1.89–12.87, p=0.001) after adjustment for gender and age. However, the CT genotype was not markedly associated with POAG (OR: 1.79; 95% CI=0.79–4.05, p=0.157). There were no marked associations with the other 25 TRPM polymorphisms studied (Table 2 and Table 3).

**Table 2:** Distributions of genotypes and alleles for the *TRPM1, TRPM2, TRPM3, TRPM4,* and *TRPM5* gene polymorphisms between the case and control groups.

Gene	Genotypes/ Alleles	Controls	n*	Cases with POAG	n*	P value
SNP						
TRPM1	CC/CT/TT	112/60/10	182	119/47/13	179	0.3399
rs28441327	C/T	284/80		285/73		0.6667
TRPM1	GG/GA/AA	112/60/10	182	117/51/10	178	0.6721
rs11070811	G/A	284/80		285/71		0.5627
TRPM1	AA/AG/GG	107/62/13	182	104/61/14	179	0.969
rs2241493 (Ser32Asn)	A/G	276/88		269/89		0.8987
TRPM2	TT/TG/GG	97/72/11	180	95/73/11	179	0.9876
rs1618355	T/G	266/94		263/95		0.9644
TRPM2	CC/CT/TT	180/1/0	182	178/0/0	178	0.4987
rs9978351 (Arg1189=)	C/T	362/2		356/0		0.4994
TRPM3	TT/TC/CC	181/1/0	182	179/0/0	179	1
rs113652718 (Val104Ala)	T/C	363/1		358/0		1
TRPM3	CC/CA/AA	132/44/6	182	128/42/9	179	0.7106
rs1328142	C/A	308/56		298/60		0.6879
TRPM4	CC/CT/TT	85/73/24	182	93/67/18	178	0.4893
rs3760663	C/T	243/121		253/103		0.2427
TRPM4	GG/GA/AA	178/4/0	182	165/10/0	175	0.1055
rs71352737 (Trp525Ter)	G/A	360/4		340/10		0.1088
TRPM5	CC/CT/TT	151/30/0	181	143/33/3	179	0.1874
rs34364959 (Gly900Ser)	C/T	332/30		319/39		0.2885
TRPM5	CC/CT/TT	55/84/42	181	55/90/31	176	0.4076
rs4929982 (Arg578Gln)	C/T	194/168		200/152		0.4286
TRPM5	CC/CT/TT	20/106/16	142	10/102/41	153	**0.0009**
rs34551253 (Ala456Thr)	C/T	146/138		122/184		0.0063
TRPM5	AA/AG/GG	65/87/30	182	68/82/28	178	0.8869
rs886277 (Asn235Ser)	A/G	214/147		218/138		0.7126
TRPM5	GG/GA/AA	100/67/15	182	96/68/12	176	0.8513
rs3986599 (Val254Ala)	G/A	267/97		260/92		0.9437

*Numbers do not always add up to total numbers because of missing values on the BioMark dynamic array system. SNP, single nucleotide polymorphism. POAG, primary open-angle glaucoma.

**Table 3:** Distributions of genotypes and alleles for the *TRPM6, TRPM7,* and *TRPM8* gene polymorphisms between the case and control groups.

Gene	Genotypes/ Alleles	Controls	n*	Cases with POAG	n*	P value
SNP						
TRPM6	GG/GA/AA	114/64/4	182	123/52/4	179	0.4588
rs3750425 (Val1388Ile)	G/A	292/72		298/60		0.3403
TRPM6	GG/GA/AA	56/73/52	181	35/88/52	175	0.0463
rs2274924 (Lys1579Glu)	G/A	185/177		158/192		0.1293
TRPM7	CC/CG/GG	179/3/0	182	179/0/0	179	0.248
rs77165588	C/G	361/3		358/0		0.249
TRPM8	CC/CT/TT	125/51/6	182	124/53/2	179	0.3646
rs1016062	C/T	301/63		301/57		0.689
TRPM8	CC/CT/TT	58/86/38	182	66/74/39	179	0.4955
rs2362294	C/T	202/162		206/152		0.6314
TRPM8	CC/CT/TT	100/67/14	181	99/63/16	178	0.8885
rs6431648	C/T	267/95		261/95		0.9603
TRPM8	AA/AG/GG	116/58/8	182	111/63/5	179	0.6113
rs2362295	A/G	290/74		285/73		0.9837
TRPM8	GG/GC/CC	125/50/7	182	129/44/6	179	0.7796
rs10803666	G/C	300/64		302/56		0.5484
TRPM8	GG/GA/AA	96/66/20	182	79/85/15	179	0.0939
rs10490018	G/A	258/106		243/115		0.427
TRPM8	GG/GA/AA	105/63/14	182	108/61/10	179	0.699
rs2052029	G/A	273/91		277/81		0.5083
TRPM8	CC/CT/TT	125/50/6	181	130/41/8	179	0.5318
rs2215173	C/T	300/62		301/57		0.7376
TRPM8	GG/GA/AA	126/50/6	182	128/42/8	178	0.621
rs6740118	G/A	302/62		298/58		0.8676

*Numbers do not always add up to total numbers because of missing values on the BioMark dynamic array system. SNP, single nucleotide polymorphism. POAG, primary open-angle glaucoma.

## Discussion

In this case-control study, we showed that the *TRPM5* gene rs34551253 (Ala456Thr) polymorphism was significantly associated with POAG and could be a risk factor for developing POAG. This is the first study to examine the association of the *TRPM* gene polymorphisms with the risk of developing POAG. Our results suggest the TT genotype of the rs34551253 (Ala456Thr) polymorphism may increase susceptibility to POAG.

Increased IOP in most cases is due to increased resistance to the outflow of aqueous humor through the trabecular meshwork, which also leads to optic nerve damage [21]. Reduction in blood flow to the optic nerve head could also be an important risk factor involved in the damage to the RGCs [22]. All eight members of the TRPM subfamily are present in vascular smooth muscle cells. Expression of all the TRPM channel proteins except TRPM5 has been reported in endothelial cells [9,11,23,24]. TRPM5 currents are Ca^2+^ and voltage dependent. In the presence of high levels of intracellular Ca^2+^, depolarization strongly increases the opening of the TRPM5 channel [9,25,26]. TRPM5 channels are blocked by an external pH of 6.0 or lower [27]. TRPM5 is highly permeable to monovalent cations and impermeable to divalent cations (i.e., Ca^2+^, Mg^2+^) [25,26,28]. This is the first study showing a marked association between TRPM5 and glaucoma. TRPM5 channel localization in ocular tissue, physiologic function of this channel, and the contribution of this channel to the glaucoma pathogenesis are currently unknown, and require further studies. In addition, the structure and function of the channel affected with the rs34551253 polymorphism is currently unknown.

Several searches have been made for associations between phenylthiourea (phenylthiocarbamide) tasting and glaucoma. Becker and Morton [29] found that the number of individuals unable to recognize the bitter taste of phenylthiourea was 28% in a “normal” eye clinic population (446 individuals) and 53% in 211 patients with primary open angle glaucoma in Caucasian patients. This finding has been further confirmed in patients with open angle glaucoma [30,31]. Becker and Ballin [32] demonstrated that 60% of the offspring of patients with POAG were non-tasters. Thus, patients with POAG are less likely to detect a bitter taste on the phenylthiourea test. The overall results show a raised incidence of non-tasters among patients with POAG.

TRPM5 is coexpressed with taste receptors, and functions as a common downstream component in sweet, bitter, and umami taste signal transduction [13,33]. Our results may suggest that *TRPM5* is a gene associated with the detection of bitter taste in patients with POAG. However, the role of the TRPM5 channel in the development of POAG is not known, and requires further studies. The *TRPM5* gene is located on chromosome 11p15.5, and our results for the TRPM gene polymorphisms add new loci of susceptibility to POAG. Our findings suggest that the *TRPM5* gene polymorphism may be involved in POAG pathogenesis.

In conclusion, this study demonstrated that a TRPM gene polymorphism is associated with POAG in the Turkish population, and the *TRPM5* polymorphism also plays a role in the pathogenesis of this glaucoma. However, the exact roles of particular TRP channels in POAG require future analysis. Further investigations of *TRPM* gene polymorphisms in different ethnic populations would be helpful in understanding the pathogenesis of POAG.

## References

[1] Resnikoff S, Pascolini D, Etya'ale D, Kocur I, Pararajasegaram R, Pokharel GP, Mariotti SP (2004). Global data on visual impairment in the year 2002.. Bull World Health Organ.

[2] Le A, Mukesh BN, McCarty CA, Taylor HR (2003). Risk factors associated with the incidence of open-angle glaucoma: the visual impairment project.. Invest Ophthalmol Vis Sci.

[3] Guo L, Moss SE, Alexander RA, Ali RR, Fitzke FW, Cordeiro MF (2005). Retinal ganglion cell apoptosis in glaucoma is related to intraocular pressure and IOP-induced effects on extracellular matrix.. Invest Ophthalmol Vis Sci.

[4] Gemenetzi M, Yang Y, Lotery AJ (2012). Current concepts on primary open-angle glaucoma genetics: a contribution to disease pathophysiology and future treatment.. Eye (Lond).

[5] Venkatachalam K, Montell C (2007). TRP channels.. Annu Rev Biochem.

[6] Montell C (2005). The TRP superfamily of cation channels.. Sci STKE.

[7] Duncan LM, Deeds J, Hunter J, Shao J, Holmgren LM, Woolf EA, Tepper RI, Shyjan AW (1998). Down-regulation of the novel gene melastatin correlates with potential for melanoma metastasis.. Cancer Res.

[8] Perraud AL, Fleig A, Dunn CA, Bagley LA, Launay P, Schmitz C, Stokes AJ, Zhu Q, Bessman MJ, Penner R, Kinet JP, Scharenberg AM (2001). ADP-ribose gating of the calcium-permeable LTRPC2 channel revealed by Nudix motif homology.. Nature.

[9] Earley S, Brayden JE (2010). Transient receptor potential channels and vascular function.. Clin Sci (Lond).

[10] Wagner TF, Loch S, Lambert S, Straub I, Mannebach S, Mathar I, Düfer M, Lis A, Flockerzi V, Philipp SE, Oberwinkler J (2008). Transient receptor potential M3 channels are ionotropic steroid receptors in pancreatic beta cells.. Nat Cell Biol.

[11] Earley S, Waldron BJ, Brayden JE (2004). Critical role for transient receptor potential channel TRPM4 in myogenic constriction of cerebral arteries.. Circ Res.

[12] Gonzales AL, Amberg GC, Earley S (2010). Ca^2+^ release from the sarcoplasmic reticulum is required for sustained TRPM4 activity in cerebral artery smooth muscle cells.. Am J Physiol Cell Physiol.

[13] Pérez CA, Huang L, Rong M, Kozak JA, Preuss AK, Zhang H, Max M, Margolskee RF (2002). A transient receptor potential channel expressed in taste receptor cells.. Nat Neurosci.

[14] Touyz RM (2008). Transient receptor potential melastatin 6 and 7 channels, magnesium transport, and vascular biology: implications in hypertension.. Am J Physiol Heart Circ Physiol.

[15] van der Wijst J, Hoenderop JG, Bindels RJ (2009). Epithelial Mg^2+^ channel TRPM6: insight into the molecular regulation.. Magnes Res.

[16] Dhaka A, Viswanath V, Patapoutian A (2006). TRP ion channels and temperature sensation.. Annu Rev Neurosci.

[17] Li Z, Sergouniotis PI, Michaelides M, Mackay DS, Wright GA, Devery S, Moore AT, Holder GE, Robson AG, Webster AR (2009). Recessive mutations of the gene TRPM1 abrogate ON bipolar cell function and cause complete congenital stationary night blindness in humans.. Am J Hum Genet.

[18] Audo I, Kohl S, Leroy BP, Munier FL, Guillonneau X, Mohand-Saïd S, Bujakowska K, Nandrot EF, Lorenz B, Preising M, Kellner U, Renner AB, Bernd A, Antonio A, Moskova-Doumanova V, Lancelot ME, Poloschek CM, Drumare I, Defoort-Dhellemmes S, Wissinger B, Léveillard T, Hamel CP, Schorderet DF, De Baere E, Berger W, Jacobson SG, Zrenner E, Sahel JA, Bhattacharya SS, Zeitz C (2009). TRPM1 is mutated in patients with autosomal-recessive complete congenital stationary night blindness.. Am J Hum Genet.

[19] van Genderen MM, Bijveld MM, Claassen YB, Florijn RJ, Pearring JN, Meire FM, McCall MA, Riemslag FC, Gregg RG, Bergen AA, Kamermans M (2009). Mutations in TRPM1 are a common cause of complete congenital stationary night blindness.. Am J Hum Genet.

[20] Nakamura M, Sanuki R, Yasuma TR, Onishi A, Nishiguchi KM, Koike C, Kadowaki M, Kondo M, Miyake Y, Furukawa T (2010). TRPM1 mutations are associated with the complete form of congenital stationary night blindness.. Mol Vis.

[21] Flammer J, Mozaffarieh M (2007). What is the present pathogenetic concept of glaucomatous optic neuropathy?. Surv Ophthalmol.

[22] Flammer J (1994). The vascular concept of glaucoma.. Surv Ophthalmol.

[23] Reading SA, Brayden JE (2007). Central role of TRPM4 channels in cerebral blood flow regulation.. Stroke.

[24] Brayden JE, Earley S, Nelson MT, Reading S (2008). Transient receptor potential (TRP) channels, vascular tone and autoregulation of cerebral blood flow.. Clin Exp Pharmacol Physiol.

[25] Hofmann T, Chubanov V, Gudermann T, Montell C (2003). TRPM5 is a voltage-modulated and Ca^2+^-activated monovalent selective cation channel.. Curr Biol.

[26] Liu D, Liman ER (2003). Intracellular Ca^2+^ and the phospholipid PIP_2_ regulate the taste transduction ion channel TRPM5.. Proc Natl Acad Sci USA.

[27] Liu D, Zhang Z, Liman ER (2005). Extracellular acid block and acid-enhanced inactivation of the Ca^2+^-activated cation channel TRPM5 involve residues in the S3–S4 and S5–S6 extracellular domains.. J Biol Chem.

[28] Prawitt D, Monteilh-Zoller MK, Brixel L, Spangenberg C, Zabel B, Fleig A, Penner R (2003). TRPM5 is a transient Ca^2+^-activated cation channel responding to rapid changes in [Ca^2+^]_i_.. Proc Natl Acad Sci USA.

[29] Becker B, Morton WR (1964). Taste sensitivity to phenylthiourea in glaucoma.. Science.

[30] Becker B, Morton WR (1964). Phenylthiourea taste testing and glaucoma.. Arch Ophthalmol.

[31] Kupfer C (1965). Open-angle glaucoma: an opportunity for early detection.. JAMA.

[32] Becker B, Ballin N (1965). Glaucoma and corticosteroid provocative testing.. Arch Ophthalmol.

[33] Ishimaru Y, Matsunami H (2009). Transient receptor potential (TRP) channels and taste sensation.. J Dent Res.

